# *Panicle Morphology Mutant 1* (*PMM1*) determines the inflorescence architecture of rice by controlling brassinosteroid biosynthesis

**DOI:** 10.1186/s12870-018-1577-x

**Published:** 2018-12-12

**Authors:** Yan Li, Xuemei Li, Debao Fu, Changyin Wu

**Affiliations:** National Key Laboratory of Crop Genetic Improvement, National Center of Plant Gene Research (Wuhan), Huazhong Agricultural University, Wuhan, 430070 China

**Keywords:** Rice, *PMM1*, Inflorescence architecture, BRs biosynthesis, *OsDWARF4*

## Abstract

**Background:**

Panicle architecture is one of the main important agronomical traits that determine branch number and grain number in rice. Although a large number of genes involved in panicle development have been identified in recent years, the complex processes of inflorescence patterning need to be further characterized in rice. Brassinosteroids (BRs) are a class of steroid phytohormones. A great understanding of how BRs contribute to plant height and leaf erectness have been reported, however, the molecular and genetic mechanisms of panicle architecture influenced by BRs remain unclear.

**Results:**

Here, we identified *PMM1*, encoding a cytochrome P450 protein involved in BRs biosynthesis, and characterized its role in panicle architecture in rice. Three alleles of *pmm1* were identified from our T-DNA insertional mutant library. Map-based cloning revealed that a large fragment deletion from the 2nd to 9th exons of *PMM1* was responsible for the clustered primary branch morphology in *pmm1–1*. *PMM1* is a new allele of *DWARF11* (*D11*) *PMM1* transcripts are preferentially expressed in young panicles, particularly expressed in the primordia of branches and spikelets during inflorescence development. Furthermore, overexpression of *OsDWARF4* (*D4*), another gene encoding cytochrome P450, completely rescued the abnormal panicle phenotype of *pmm1–1*. Overall, it can be concluded that *PMM1* is an important gene involved in BRs biosynthesis and affecting the differentiation of spikelet primordia and patterns of panicle branches in rice.

**Conclusions:**

*PMM1* is a new allele of *D11*, which encodes a cytochrome P450 protein involved in BRs biosynthesis pathway. Overexpression of *D4* could successfully rescue the abnormal panicle architecture of *pmm1* plants, indicating that *PMM1/D11* and *D4* function redundantly in BRs biosynthesis. Thus, our results demonstrated that *PMM1* determines the inflorescence architecture by controlling brassinosteroid biosynthesis in rice.

**Electronic supplementary material:**

The online version of this article (10.1186/s12870-018-1577-x) contains supplementary material, which is available to authorized users.

## Background

Inflorescence architecture and spikelet formation are unique features of grasses such as rice, maize and wheat. In rice, inflorescence (also called panicle) architecture can be categorized into nine successive stages according to the morphological dynamic changes [1]. During the transition from vegetative to reproductive phase, inflorescence meristem (IM) firstly initiates from shoot apex meristem (SAM), and subsequently produces primary, secondary, and sometimes a higher order of panicle branch meristem (BM). On lateral panicle branches, spikelet meristem (SM) initiates and subsequently bears florets and finally develops into grain [1–3]. As a result, the length of the main axis as well as the number/length of primary and secondary inflorescence branches are two major determinants for the number of spikelets per panicle and yield of rice [4].

In recent years, multiple genes have been identified to determine inflorescence development processes and finally form the morphology of panicle. *SMALL PANICLE* (*SPA*), *REDUCED CULM NUMBER1* (*RCN1*), *LAX PANICLE1* (*LAX1*), and *LAX2* are involved in the initiation of BM and SM [5–9]. *ABERRANT PANICLE ORGANIZATION1* (*APO1*), *APO2/RFL* and *TAWAW1* (*TWA1*) are responsible for the identity of BM by preventing precocious conversion of BM to SM [10–16]. *FRIZZLE PANICLE* (*FZP*) is required for floral organ initiation and identity through preventing formation of axillary meristems of rice spikelets [17–19]. *TILLERS ABSENT1* (*TAB1*) has been shown to be involved in the activity of axillary meristems in rice [20, 21]. Some other genes, such as *GRAIN NUMBER1* (*Gn1a*), *DENSE AND ERECT PANICLE1* (*DEP1*), *DEP2* and *DEP3*, showed effects on the number of branches or spikelets in rice [22–27]. *SHORT PANICLE1* (*SP1*), *ABERRANT SPIKELET AND PANICLE1* (*ASP1*), *PANICLE APICAL ABORTION1* (*PAAB1*), *TUTOU1* (*TUT1*) and *SQUAMOSA PROMOTERBINDING PROTEIN-LIKE 6* (*SPL6*) have been identified to be involved in the elongation of inflorescence branches and degeneration of panicles in rice [28–32]. Besides, a few genes involved in panicle density have also been reported. *OsLG1/SPR3* encodes a SBP-domain transcription factor and regulates a closed panicle trait, a selected trait during rice domestication [33–35]. In addition, *CL* and *CL*-*DZ* have been described as genes that lead to typical clustered spikelets and are necessary for the formation of bract primordia in the primary and secondary branch meristems [36, 37]. Our previous investigation has identified a *panicle morphology mutant 1* (*pmm1*), which also causes a clustered primary branches in panicle [38]. *CLUSTERED PRIMARY BRANCH1* (*CPB1*) was identified to influence the development of panicle architecture, leaf angle and seed size [39]. Despite of these advances, the molecular and genetic mechanisms underlying the differentiation of spikelets or branch meristems are still poorly understood.

Brassinosteroids (BRs) are a class of steroid phytohormones, which influence both plant height and leaf erectness in rice [40]. So far, several genes playing crucial roles in BRs biosynthesis or signaling pathways have been identified via mutants either BR-deficient or BR-insensitive. The BR-insensitive mutants showed no significant response to the exogenous application of BRs due to the loss-of-function of the genes involved in the signal transduction pathway for BRs, such as *OsBRI1/DWARF61*, *DWARF1*, *OsBZR1*, *14–3-3*, *OsBAK1*, *OsBRL*, *SG1*, *XIAO*, *DWARF62*, and *OsGRAS19* [41–50]. BR-deficient mutant, such a*s dwarf2 (d2)*, *dwarf4* (*d4*), *dwarf11* (*d11*), *brd1* and *brd2*, showed defects in BR biosynthesis, but the defects could be rescued by exogenous application of BRs [51–57]. Both *DWARF4* (*D4*) and *DWARF11* (*D11*) encode a cytochrome P450*,* which involved in BRs biosynthesis and have been characterized to control the plant architecture [39, 54]. *D4* encodes CYP90B and catalyzes C-22 hydroxylation, which is a rate-limiting step of BRs biosynthesis [58, 59]. Mutation of *D4* caused defects in rice plant morphology, such as a slight dwarfed stature and more erect leaves, indicating that *D4* may mainly function during vegetative organs in rice [54]. *D11*, which encodes CYP724B1, is essential to maintain the levels of bioactive BRs synthesis [55]. *d11* mutant showed the pleiotropic defective morphologies including erect leaves, shortened internodes and small seeds [55]. However, the expression patterns of *D11* and *D4* and their roles in panicle architecture need to further elucidate.

In this study, we identified two additional alleles of *pmm1*, which exhibited a clustered primary branches phenotype during panicle development. Our genetic mapping results revealed that *PMM1* is a new allele of *D11*, which encodes a cytochrome P450 protein involved in BRs biosynthesis pathway. Transgenic analysis indicated that *PMM1/D11* and *D4* function redundantly in BRs biosynthesis, indicating that BRs biosynthesis is required for the panicle architecture in rice.

## Results

### Identification of *pmm1* mutants

**Fig. 1:**
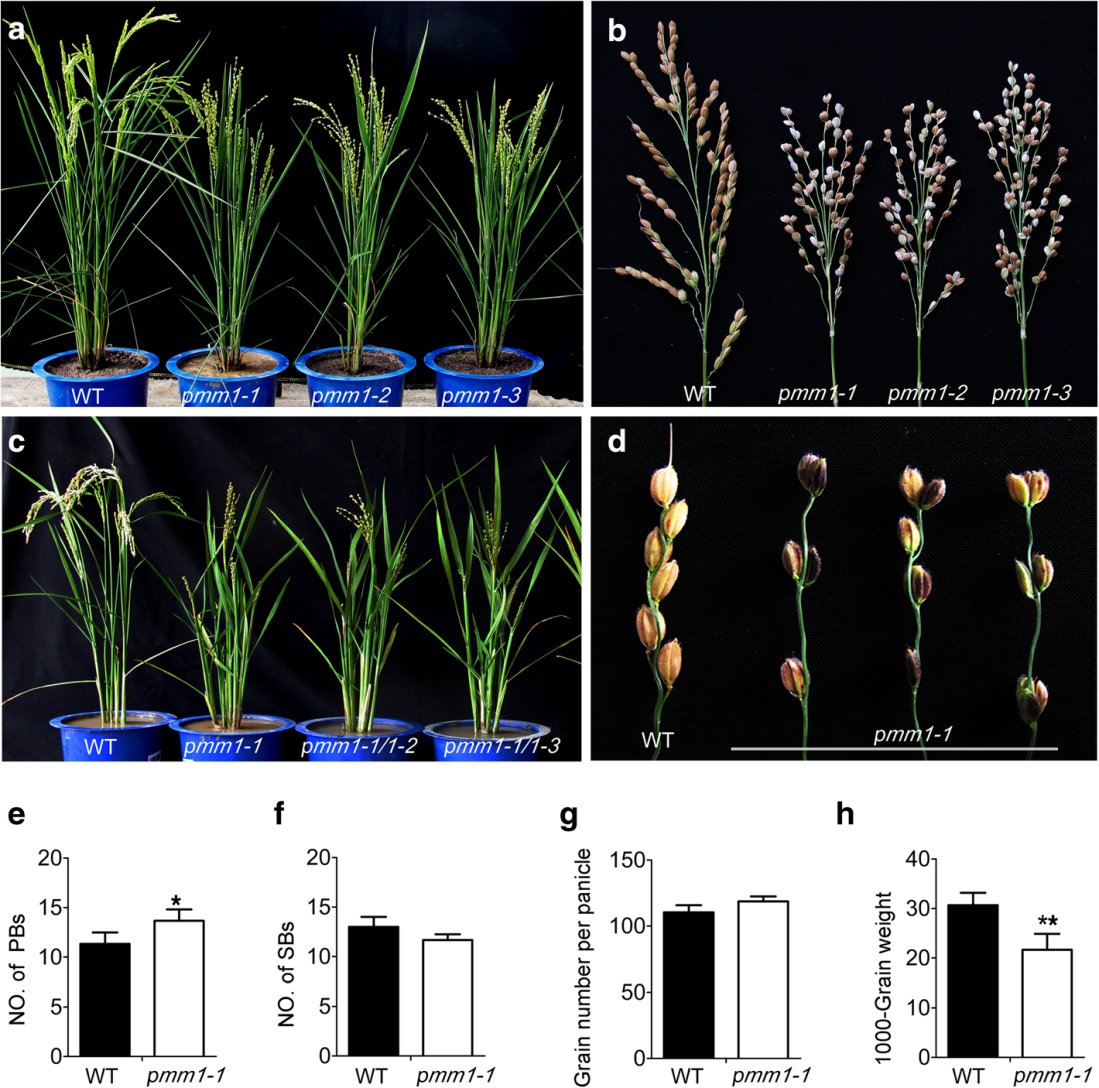
Phenotypic characterization of *pmm1* mutants. **a** Plant morphology between WT (ZH11) and three alleles of *pmm1* mutants. **b** Panicle architecture between WT and three alleles of *pmm1* mutants. **c** Plant morphology of F1 plants of *pmm1–1/pmm1–2* and *pmm1–1/pmm1–3*. **d** Spikelet morphology between WT *pmm1–1* mutants. **e**-**h** Comparison of the primary branch number (PBs) (**e**), secondary branch number (SBs) (**f**), grain number per panicle (**g**) and 1000-grains weight (**h**) between WT and *pmm1–1*. Data are shown as means ± SE (*n* = 10). Significant at **P < 0.01 and *P < 0.05

Previously, we have identified a *panicle morphology mutant 1* (*pmm1*) [38], which showed a slight phenotype in vegetative growth (Fig. 1a) but a strong morphological defects in inflorescence architecture (Fig. 1b). We designated this panicle morphology mutant as *pmm1–1*. The panicle morphology of *pmm1–1* was dramatically altered compared with that of wild type (WT), such as clustered primary branching, opposite grains, and small grains (Fig. 1b). In order to collect more panicle morphology mutants, we screened our T-DNA insertional mutant library [60] and identified two additional lines, which showed obvious clustered primary branches similar to *pmm1–1* (Fig. 1a, b). Genetic analyses showed that about one- quarter of their progenies of the heterozygous were defect panicle morphology and others showed normal panicle, indicating that the clustered primary branching of each mutant was controlled by a single recessive allele, respectively. We did not detect any phenotypic segregation when crossing each mutant with *pmm1–1* (Fig. 1c), suggesting that the mutation loci in these two independent lines were allelic to that in *pmm1–1.* Thus, these two lines were designated as *pmm1–2* and *pmm1–3*, respectively. We chose *pmm1–1* for further examination.

### Characterization of pmm1

Next, we characterized the morphological defects of *pmm1* compared with WT. In the paddy field, all the *pmm1* plants (*pmm1–1*, *pmm1–2* and *pmm1–3*) appeared slightly dwarf and produced erected leaves (Fig. 1a). After heading, *pmm1* plants showed obvious multiple morphological defects in panicle, such as clustered panicle, shortened axis, clustered primary branches and opposite spikelets (Fig. 1b, d). We further compared the yield traits between *pmm1–1* and the WT plants under normal growth conditions. The primary branches number per panicle was increased in *pmm1–1* (Fig. 1e), while as for the other traits, no obvious differences concerning secondary branches number per panicle and grain number per panicle in *pmm1–1* compared with WT (Fig. 1f, g). A 30% decrease in 1000-grain weight was decreased in *pmm1–1* (Fig. 1h).

**Fig. 2:**
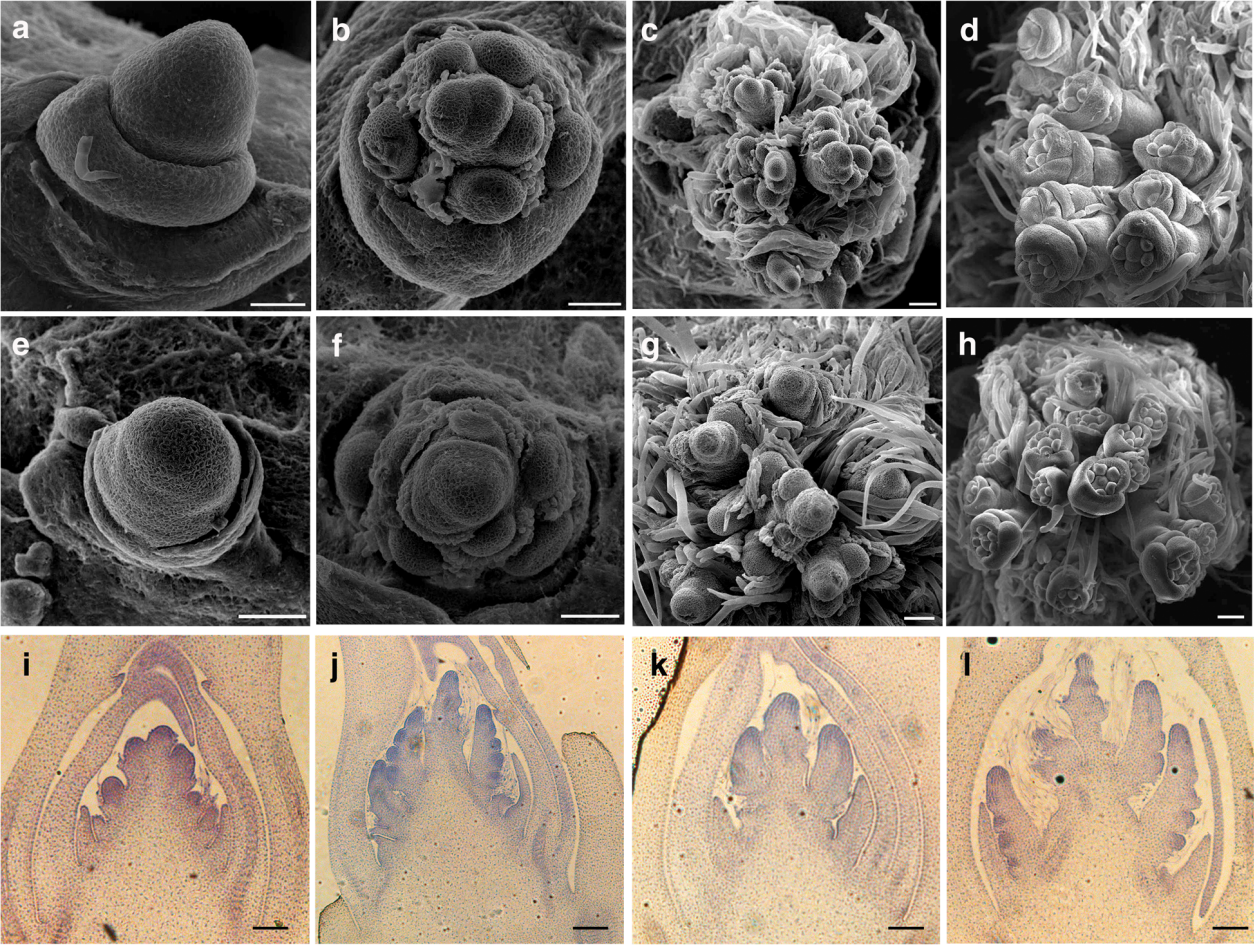
Microscopic observation of the developing panicles in WT and *pmm1–1.* Scanning electron microscopy (SEM) analysis of young panicle development between WT (**a**-**d**) and *pmm1–1* (**e**-**h**) at the vegetative stage (**a**, **e**), the primary branch primordial formation stage (**b**, **f**), the second primordial formation stage (**c**, **g**), the spikelet primordial formation stage (**d**, **h**). Longitudinal section analysis of young panicle development in WT (**i**, **j**) and *pmm1–1* (**k**, **l**) at the primary branch primordial formation stage (**i**, **k**) and the second primordial formation stage (**j**, **l**). Bars = 100 μm

To better characterize the panicle developmental defects of *pmm1–1*, we compared the panicle development between WT and *pmm1–1* using scanning electron microscopy (SEM). No remarkable morphological differences in apices were observed between *pmm1–1* and WT (Fig. 2a, e). At the reproductive stage, the primary branches were generated normally in WT (Fig. 2b). However, the primary branch primordia in *pmm1–1* seemed to generate in a whorl pattern (Fig. 2f). At the secondary branch primordial formation stage, no obvious differences were observed between *pmm1–1* and WT (Fig. 2c, g). At the spikelet formation stage, the spikelet primordia were normally developed in WT (Fig. 2d) and *pmm1–1* (Fig. 2h). Compared to WT (Fig. 2i, j), histological analysis revealed that such malformation would due to the clustered primary branches pattern developed in *pmm1–1* (Fig. 2k, l). These observations suggested that the generation pattern of primary branches were altered in *pmm1–1* compared with WT*.*

### Identification of PMM1

**Fig. 3:**
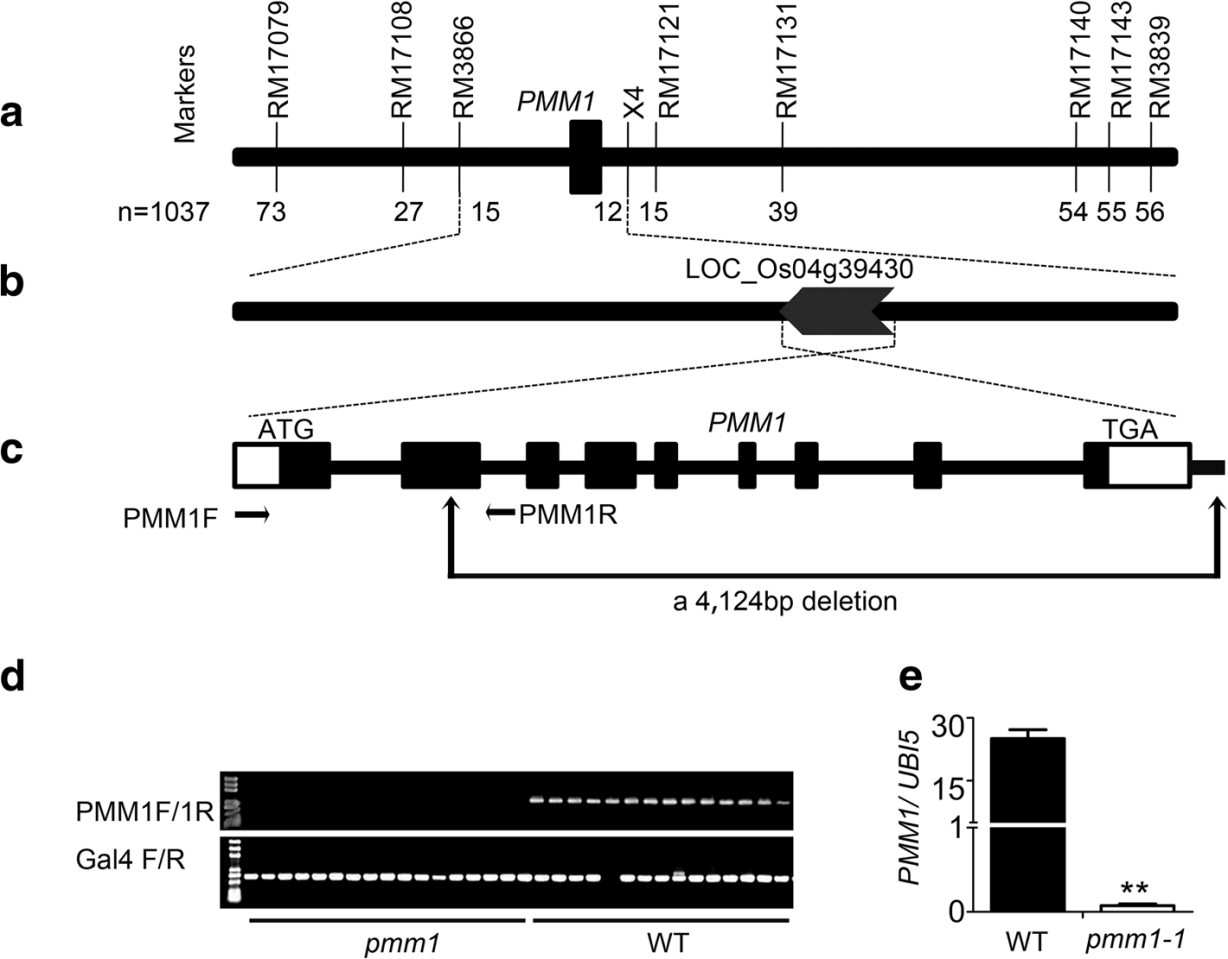
Map-based cloning of *PMM1*. **a** Linkage map of *PMM1* on chromosome 4. The number of recombinants between the molecular marker and *PMM1* is indicated. **b** Location of *PMM1*. **c** Gene structure of *PMM1*. The black boxes represent the exons and lines between the boxes represent introns. The deletion fragment in *pmm1–1* is shown. **d** Genotyping of ZH11 and *pmm1–1* by PCR analysis. **e** Transcript levels of *PMM1* in WT and *pmm1–1* determined by qRT-PCR. Rice *UBIQIUTIN5* was used as an internal control. Data are presented as means ± SE (*n* = 3). Significant at **P < 0.01

Previously, we have already generated a F2 mapping population by crossing *pmm1–1* with an *indica* variety ZS97 [38] and delimited *PMM1* to a 147 kb region between the two markers RM3866 and X4 (Fig. 3a). Since no more polymorphic markers could be developed to further narrow down the candidate locus (Additional file 1: Table S1), we sequenced and analyzed all the 21 predicted genes in this region (Additional file 2: Table S2). Finally, we identified a 4124 bp deletion in this region (Fig. 3b), which resulted in the deletion of a large fragment from 2nd to 9th exons of LOC_Os04g39430 (*D11*) (Fig. 3c). Thus, *pmm1–1* mutant might be a knock-out mutant of *D11*. Further analysis showed that the alteration of panicle architecture in *pmm1–1* mutant were not associated with the T-DNA insertion (Fig. 3d), suggesting that the large fragment deletion might have occurred due to tissue culture process. qRT-PCR results revealed that the transcripts of *D11* were significantly blocked in the young panicles of *pmm1–1* compared with WT (Fig. 3e). Therefore, *D11* is likely to be a candidate for *PMM1*.

### Genetic complementation of *pmm1*

**Fig. 4:**
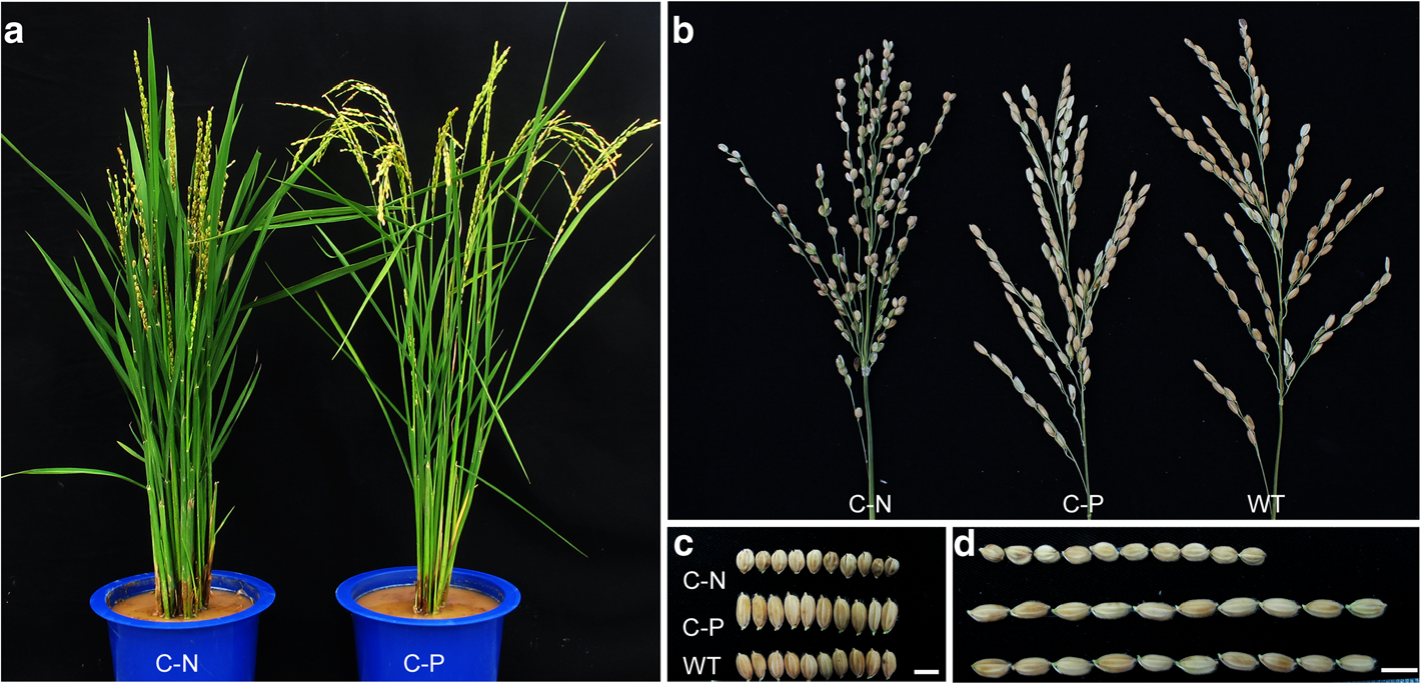
Genetic complementation of *pmm1–1*. **a** Comparison of plant phenotypes between negative and positive transgenic plants. **b** Panicle architecture of transgenic plants. **c** Grain width of transgenic plants. **d** Grain length of transgenic plants. C-N, negative transgenic plants; C-P, positive transgenic plants. Bars = 0.5 cm

To confirm that *D11* is responsible for the phenotype of *pmm1–1*, we generated a vector containing the *D11-*coding region under the control of its native promoter and transformed it into *pmm1–1* mutant by *Agrobacterium tumefaciens*-mediated transformation [60]. Forty-seven independent transgenic plants were generated, and the clustered primary branches of *pmm1–1* were completely restored to normal inflorescence architecture in all the transgenic positive plants (Fig. 4a, b). Besides, the grain length and grain width were increased compared to the negative plants (Fig. 4c, d), the grain size also enlarged as much as that in WT. Collectively, these results suggest that the mutation in *D11* is responsible for abnormal panicle morphology of *pmm1–1*.

### PMM1 encodes a cytochrome P450 involved in BRs biosynthesis

**Fig. 5:**
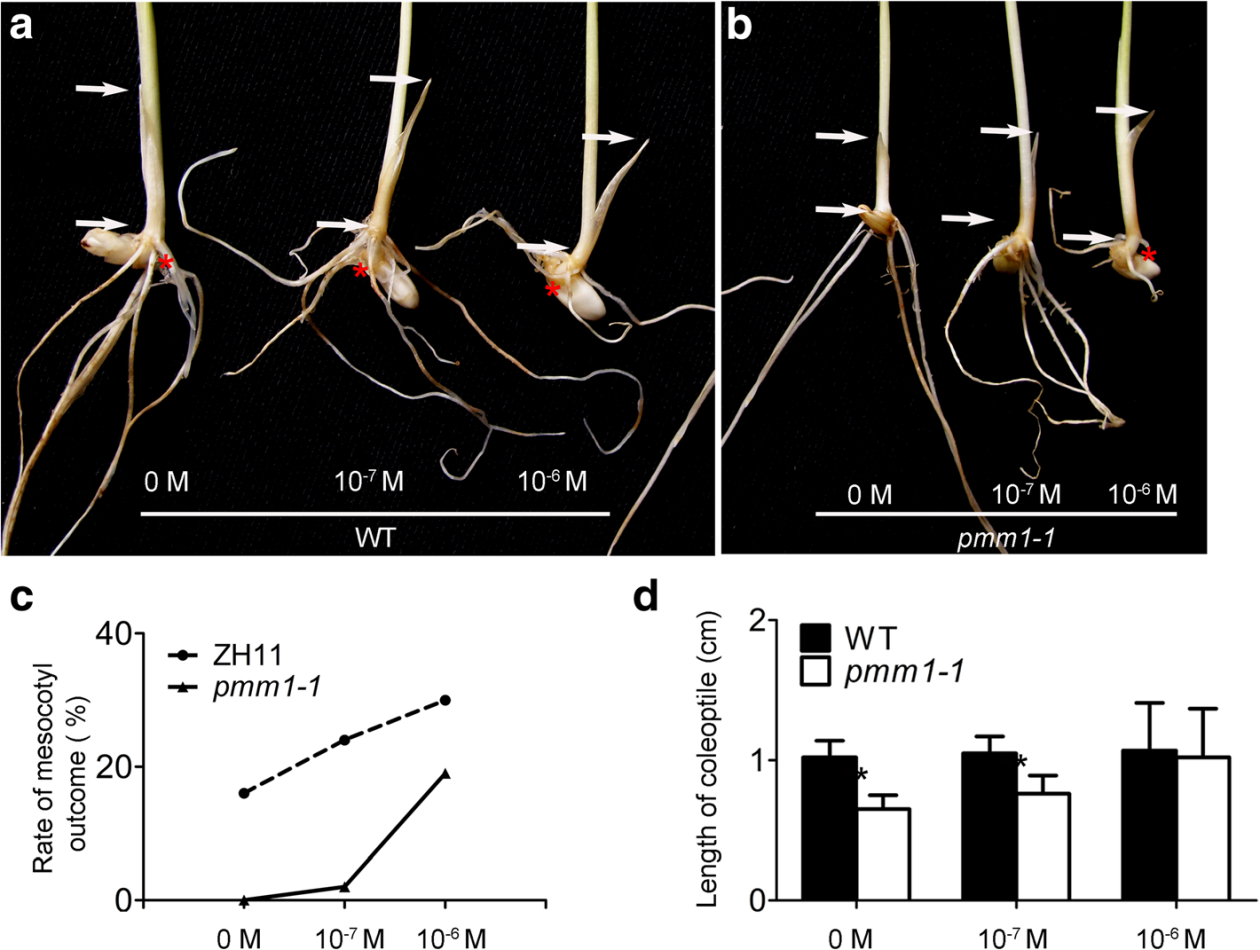
Response of seedlings to BL. **a** Skotomorphogenesis of WT plants treated with BLs. **b** Skotomorphogenesis of *pmm1–1* plants treated with BLs. **c** Rate of mesocotyls emergence between WT and *pmm1–1* plants. **d** Length of coleoptiles between WT and *pmm1–1* plants. The positions of mesocotyls in these plants are indicated by red stars. The lengths of coleoptile in these plants are indicated by white arrows. Data are presented as means ± SE (*n* = 10). Significant at **P < 0.01

*D11* encodes a cytochrome P450 superfamily protein CYP724B1, facilitating the supply of 6-DeoxoTY and TY during BRs biosynthesis [55]. As *PMM1* was identified as a new allele of *D11*, we speculated that *pmm1* would be deficient in BRs biosynthesis and sensitive to BRs. To confirm that, we carried out morphological examination of *pmm1–1* in the complete darkness. The emergence rate of mesocotyls and the length of coleoptile were investigated in *pmm1–1* and WT plants. In the dark, the WT plants showed an obvious skotomorphogenic phenotype including elongated mesocotyls and coleoptile (Fig. 5a). However, the mesocotyls were not elongated and the length of coleoptile was short in *pmm1–1* (Fig. 5b). Then, we treated *pmm1–1* plants with the most bioactive BRs compound BL in darkness. The emergence rate of mesocotyls in *pmm1–1* was increased with improving the concentration of BL (Fig. 5c). The length of coleoptile in *pmm1–1* was almost the same as that in WT under the concentration of 10^− 6^ M (Fig. 5d). Our result confirmed that *pmm1–1* is a BR-sensitive mutant and *PMM1* might be involved in BRs biosynthesis.

### PMM1 transcripts are abundant in young panicle

**Fig. 6:**
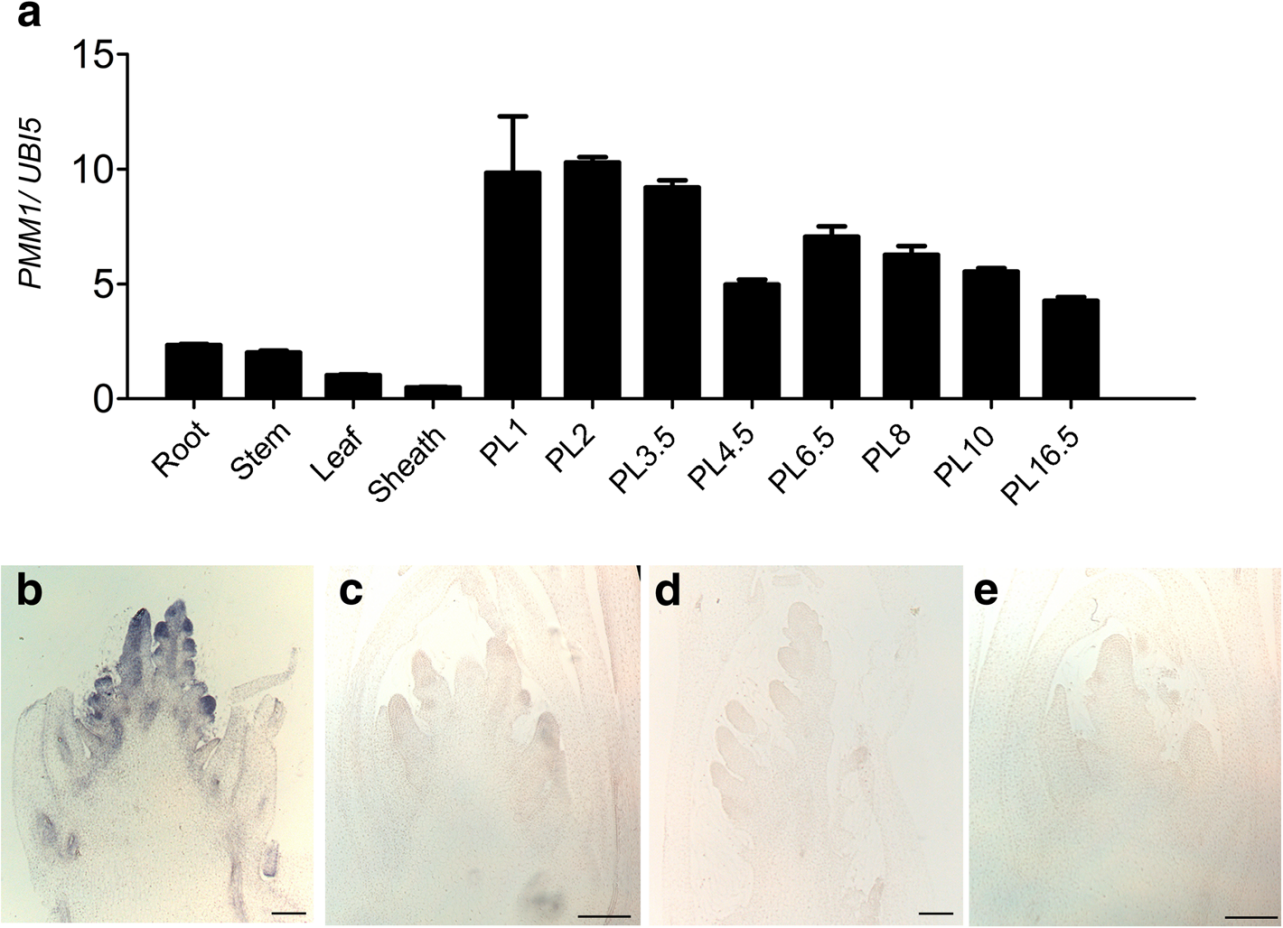
Expression pattern of *PMM1*. **a** Expression profiles of *PMM1* in the root, stem, leaf, leaf sheath and developing panicles at the 1, 2, 3.5, 4.5, 6.5, 8, 10 and 16.5-cm stages before heading. Rice *UBIQIUTIN5* was used as an internal control. Data are presented as means ± SE (*n* = 3). **b**-**e** In situ hybridization assay of *PMM1* in young panicles of WT (**b**) and *pmm1–1* (**c**). Negative controls with sense probe in in young panicles of WT (**d**) and *pmm1–1* (**e**). Bars = 100 μm

To examine the *PMM1* expression pattern, we firstly conducted RT-PCR assays on the RNA samples from root, stem, leaf blade, leaf sheath and developing panicles. *PMM1* transcripts were detected at a very low level in the examined vegetative organs, but highly accumulated in the developing panicles (Fig. 6a). Subsequently, in situ hybridization was carried out to determine the precise expression pattern in the young panicles. The RNA in situ hybridization signals was obviously detected in the branch primodia and the spikelet primodia in the developing young panicle (Fig. 6b). However, the *PMM1* signals were absent in *pmm1–1* (Fig. 6c). Using the sense-strand probes, no signal was detected either in *pmm1–1* (Fig. 6d) or in WT (Fig. 6e). Because *PMM1* preferentially expressed in young panicle and its mutant showed panicle defects, we deduced that *PMM1* would be required for the panicle architecture in rice.

### Overexpression of OsDWARF4 could rescue the abnormal panicle architecture of pmm1

*OsDWARF4* (*D4*) has been shown to function redundantly with *D11* in BRs biosynthesis [54]. In order to examine the roles of *D4* in the panicle architecture in rice, we firstly conducted the sequences comparison between D4 and D11. Sequence alignment showed that the amino acid sequence of D11 shared more than 40% identity with D4 (Additional file 3: Figure S1a). Then, we performed qRT-PCR analysis to examine the expression patterns of *D4* in different tissues of both *pmm1–1* and WT. The results showed that *D4* was highly expressed in the root, culm, leaf and leaf sheath but lowly expressed in the developing panicle (Additional file 3: Figure S1b). The transcript levels of *D4* were increased in *pmm1–1* plants, suggesting that *D4* expression might be feedback-regulated when loss of function of *PMM1*/*D11*.

**Fig. 7:**
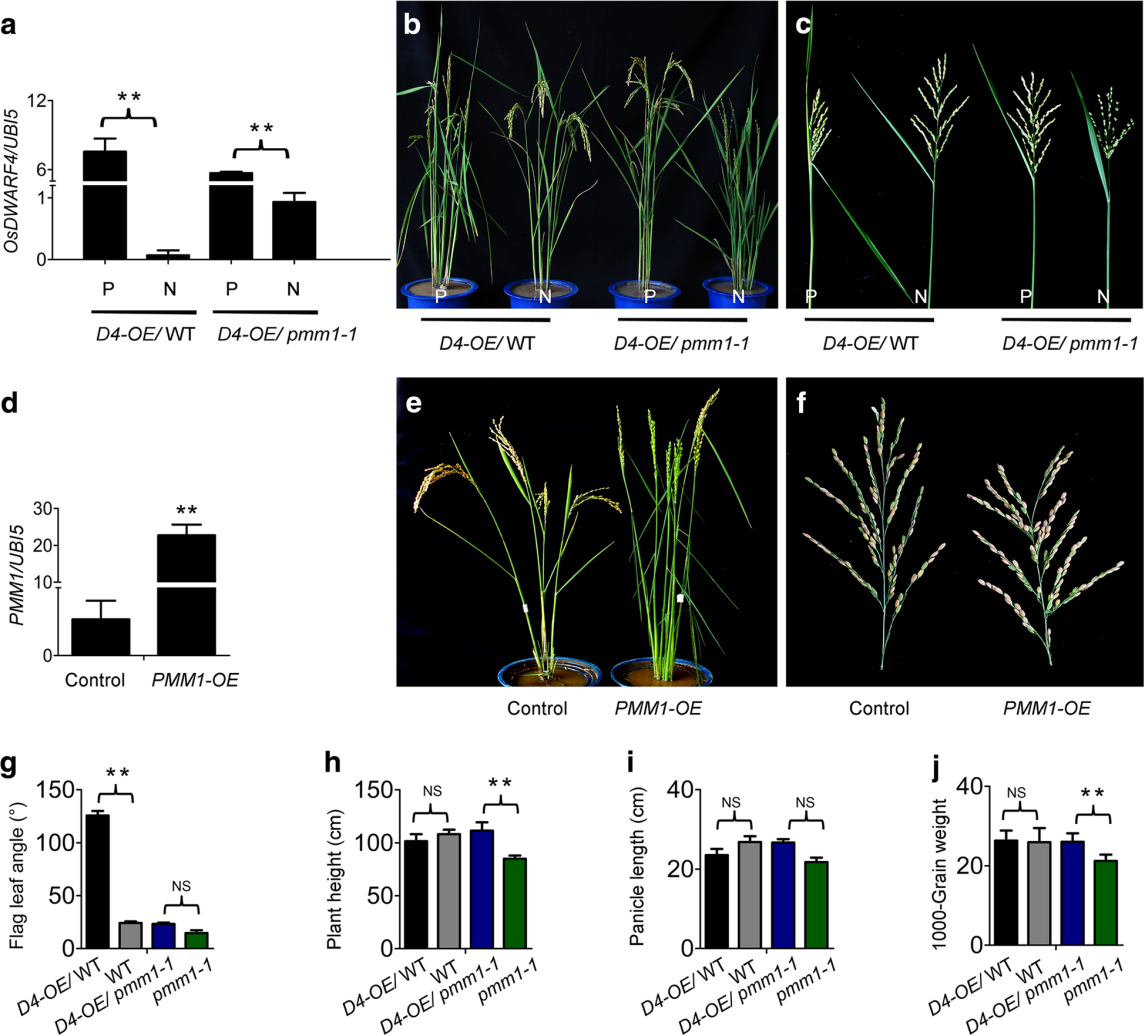
Morphological characterization of *D4*-overexpressing and *PMM1*-overexpressing plants. **a** Transcript levels of *D4* in positive (P) and negative (N) transgenic plants in WT and *pmm1–1* backgrounds, respectively. Rice *UBIQIUTIN5* was used as an internal control. Data are presented as means ± SE (*n* = 3). **b** Plant morphology of *D4*-overexpressing lines. **c** Panicle morphology of *D4*-overexpressing lines. **d** Transcript levels of *PMM1* in positive (P) and negative (N) transgenic plants. Rice *UBIQIUTIN5* was used as an internal control. Data are presented as means ± SE (*n* = 3). **e** Plant morphology of *PMM1*-overexpressing lines. **f** Panicle morphology of *PMM1*-overexpressing lines. **g** Flag leaf angles of *D4*-overexpressing lines. **h** Plant height of *D4*-overexpressing lines. **i** Panicle length of *D4*-overexpressing lines. **j** 1000-grains weight of *D4*-overexpressing lines. Data are presented as means ± SE (*n* = 3). Significant at **P < 0.01

To confirm the functional redundancy between *D4* and *PMM1*/*D11*, we generated 30 plants overexpressing *D4* in *pmm1–1* and in Zhonghua 11 (control), respectively. qRT-PCR analysis confirmed the positive transgenic lines (D4-OE, D4-OE/ *pmm1–1*) had overexpression of *D4* in young panicles compared with the negative transgenic lines (Fig. 7a). *D4*-overexpressing plants (e.g. D4-OE #8) exhibited larger leaf angles, a typical phenotypic characteristic of excess BRs (Fig. 7b). In the plants of D4-OE/*pmm1–1* (#13)*,* plant height and the abnormal panicle morphology could be completely recovered by overexpression of *D4* expression in *pmm1–1* (Fig. 7b, c). We have also generated 48 plants overexpressing *PMM1*/*D11*. qRT-PCR analysis confirmed the overexpression of *PMM1* in the independent positive transgenic lines compared with the negative transgenic lines (Fig. 7d). The plants overexpressing *PMM1* (#12) displayed large leaf angles (Fig. 7e, g) and increase in plant height (Fig. 7e, h), but a decrease in panicle length (Fig. 7f, i). We have detected a slight increase grain number per panicle and 1000-grain weight in *PMM1* overexpression lines compared with control (Fig. 7j). Taken together, our result suggested that *D4* and *PMM1*/*D11* function redundantly in biosynthesis of BRs, which is crucial for the normal panicle architecture development.

### PMM1 may participate in multiple biological processes

**Fig. 8:**
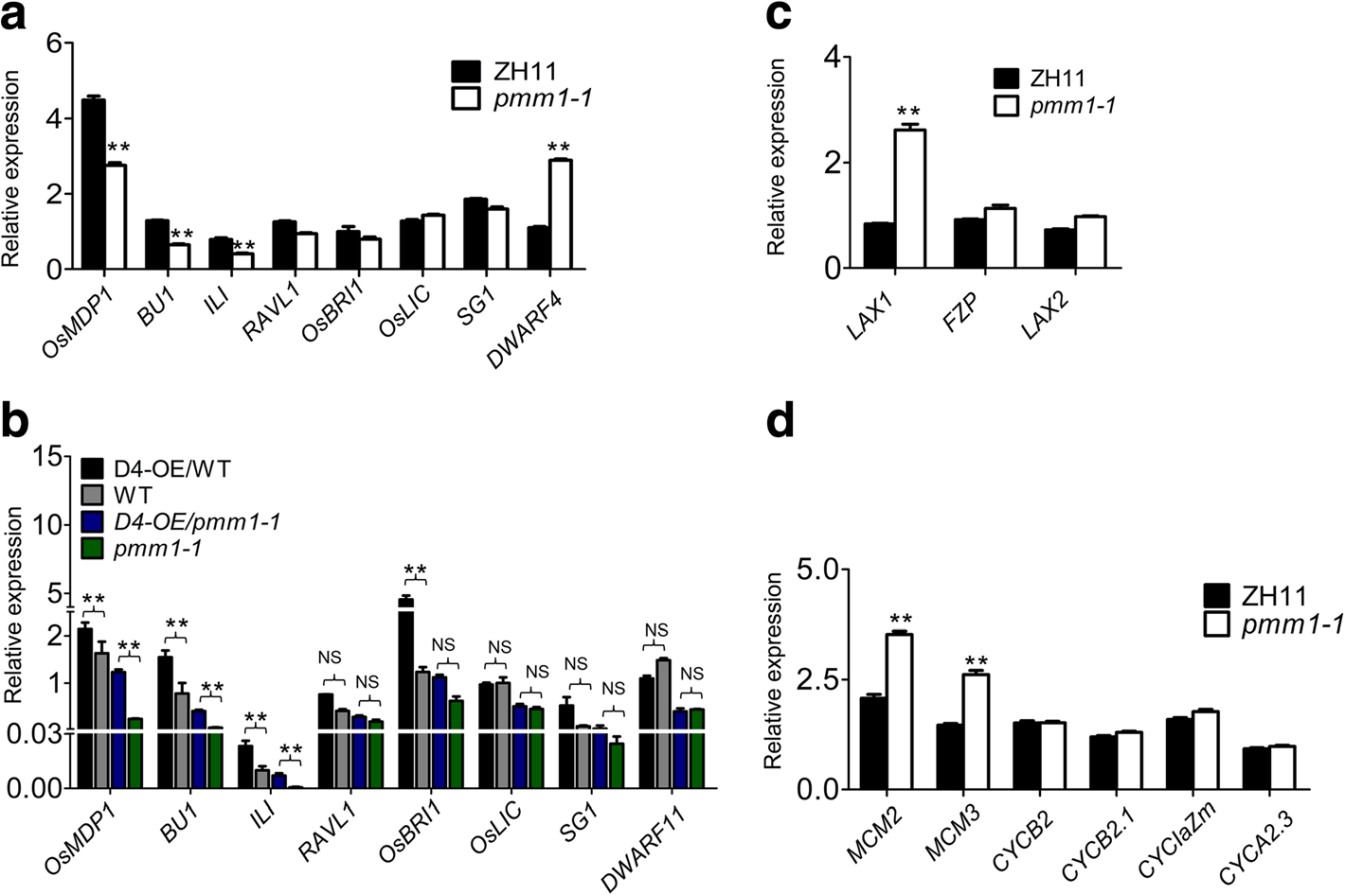
Expression pattern of corresponding genes in the young panicles of WT, *pmm1–1* and *D4*-overexpressing plants. **a** Expression pattern of BR-related genes in WT and *pmm1–1* plants. **b** Expression pattern of BR-related genes in *D4*-overexpressing plants. **c** Expression levels of several genes related to panicle development in WT and *pmm1–1* plants. **d** Expression levels of several genes related to cell cycling. Rice *UBIQIUTIN5* was used as an internal control. Data are presented as means ± SE (*n* = 3). Significant at **P < 0.01 and *P < 0.05

Previous works have elucidated that *PMM1/D11* encodes a cytochrome P450 involved in BRs biosynthesis process [54, 55]. In order to investigate the possible roles of *PMM1* in BR biosynthesis, we examined the expression levels of one BR biosynthesis gene (*D4*) and seven BR signal transduction genes (*OsMDP1*, *BU1*, *ILI1*, *RAVL1*, *OsBRI1*, *OsLIC* and *SG1*) in the young panicles of *pmm1–1* by qRT-PCR. Compared to that of WT, the expression levels of BR-signaling genes did not altered significantly in *pmm1–1* except for *MDP1*, which was significantly reduced in *pmm1–1* (Fig. 8a). As reported in previous literature, the expression level of the BRs biosynthesis gene *D4* was significantly increased in *d11/pmm1* (Fig. 8a), indicating that a feedback regulation of BRs biosynthesis genes may be trigged in *d11/pmm1* [54]. In the D4-OE/WT plants, the expression levels of some BR signal transduction genes (such as *OsBRI1*, *OsMDP1*, and *BU1*) showed dramatically increased compared to the WT (Fig. 8b). These results further indicated that *D11* and *D4* might be responsible for BR biosynthesis in different organs. Although overexpression of *D4* in the young panicles could rescue the clustered panicles in *pmm1*, the dosage effect of BRs might cause the different expression patterns of BR signal transduction genes in *D4-OE/pmm1* plants (Fig. 8b).

*LAX1*, *LAX2* and *FZP* are classic genes, which determine the inflorescence architecture by controlling rachis-branch meristem development [6, 9, 17]. We have examined whether the panicle architecture regulatory pathway is associated with the *PMM1/D11* using qRT-PCR analysis in young panicle. The expression levels of *FZP* and *LAX2* were not significantly changed in *pmm1–1*, suggesting that *PMM1* does not affect the expression of *FZP* and *LAX2* (Fig. 8c). In contrast, the expression of *LAX1* was significantly up-regulated in *pmm1–1*, indicating that BRs might be involved in *LAX1* regulatory pathways which influence panicle formation in rice.

Previous studies have suggested that BRs can influence seed sizes by affecting cell cycling genes in rice [49]. In this study, mutation in *PMM1* also caused small and round grains compared with WT (Fig. 1b). Then, we analyzed the expression levels of six cell cycle-related genes (*MCM2*, *MCM3*, *CYCB2*, *CYCB2*.1, *CYClaZm*, *CYCA2*.3) in young panicles of *pmm1–1* and WT. The expression of both *MCM2* and *MCM3* significantly increased in *pmm1–1* compared with that in WT, but that of the other cell cycle-related genes (*CYCB2*, *CYCB2*.1, *CYClaZm*, *CYCA2*.3) were not significantly changed (Fig. 8d). These results suggested that *PMM1*, a BRs biosynthesis gene, might contribute a possible connection between BRs and cell cycle regulation in controlling grain size in rice.

## Discussion

### Mutation in PMM1 resulted in clustered panicles

Panicle morphology is a critical determinant of grain yield in rice and other grain crops. So far, diverse inflorescence architectures have been identified in rice, such as short panicle [28], long panicle [61], large panicle [62], lax panicle [6, 7, 9], frizzy panicle [17–19], dense panicle [23–25], abortive panicle [29–31], spreading panicle [33–35], and clustered panicle [39], which would be potential genetic resources for breeding of an ideal rice panicle morphology. Identification of superior alleles for panicle architecture and unraveling their molecular basis would shed light on future breeding program.

In this study, we identified 3 independent lines with mutations in *pmm1* exhibiting whorled primary branches and clustered spikelets (Fig. 1c, d). SEM results showed that the clustered branch precordia occurred during the young panicle development in *pmm1–1* (Fig. 1). Therefore, we hypothesized that *PMM1* plays an important role in determination of branch and spikelet primordium formation in inflorescences. In addition, expression data showed that *PMM1* was predominantly expressed in developing young inflorescence, particularly in branches and spikelet primordia (Fig. 6b). We speculate that *PMM1* largely affects the panicle growth and development in the early stages of axillary meristem initiation. Considering *pmm1–2* and *pmm1–3* are allelic to *pmm1–1*, we have not further characterized their panicle morphology in detail. Actually, by screening our T-DNA insertion mutant library, we have collected more than 50 independent mutant lines that showed obvious altered panicle architecture. However, only these 3 independent lines (*pmm1–1*, *pmm1–2*, and *pmm1–3*) caused an obvious clustered primary branches in panicle. Previous literature have reported that *CPB1* gene, an allele of *DWRAF11*, also controls panicle architecture and seed size in rice [39]. Mutation in *DWRAF11* resulted in remarkable clustered branches in panicles, indicating that BR biosynthesis in panicle is essential for normal inflorescence architecture in rice.

### PMM1 encodes a cytochrome P450 related to BR biosynthesis

Physiological studies have demonstrated that most BR-biosynthesis genes are involved in diverse processes during plant development, such as stem elongation, leaf angle, tiller number, plant height, male fertility, senescence and biotic/abiotic stresses [63, 64]. In this study, loss function of *PMM1* also resulted in a typical phenotype of BR biosynthesis deficiency, such as compact plant type with reduced leaf angle, small and round grains (Fig. 1b, d). Actually, we also observed the reduced leaf angles and shortened internodes in *pmm1–1* compared to WT (Additional file 4: Figure S2 a-g). Additionally, the expression levels of genes associated with lamina joint inclination and BR-related genes associated with internode elongation have been changed in *pmm1–1* plants (Additional file 4: Figure S2 h, i). The *pmm1–1* plants showed a highly sensitivity to BLs treatment (Fig. 5). These results strongly suggest that *PMM1* encodes a cytochrome P450 and participates in BR biosynthesis pathway [55].

Besides the typical characteristic phenotypes of BR deficiency, the *pmm1–1* mutant, with the deletion of a large fragment from 2 to 9 exons, displayed a novel phenotype of whorled branches and clustered spikelets, with 2–3 abnormal spikelets clustering on each panicle branch (Fig. 1d). *PMM1* is a novel allele of *DWARF11*. Previous studies found that some allelic *D11* mutations were generated because of single nucleotide deletion (*d11–1*), insertion (*d11–2*) or substitution (*d11–3*, *d11–4* and *cpb1*) [39, 55]. Nevertheless, none of these mutants exhibited whorled primary branches and clustered spikelets, except for that *cpb1* showed clustered primary branches but no obvious clustered spikelets [39]. These results suggest that *Oryza sativa* cytochrome P450 family member CYP724B1 affects plant architecture, especially inflorescence architecture, might be in a transcript dosage-dependent manner, or depending on rice varieties.

Many other kinds of BR-related genes have also been demonstrated to affect rice plant architecture, such as *BRD1*, *BRD2* and *XIAO* [49, 51, 53, 56]. Among them, *XIAO* was reported to be involved in the control of organ size by cell cycling. We have observed the small and round grains in three *pmm1* mutants (Fig. 1b). Expression analysis showed that there were no significant changes in gene expression between WT and *pmm1–1*, except for *MCM2* and *MCM3* (Fig. 8d). These results suggest that *PMM1/D11* controls the seed sizes possibly through cell cycling as well.

### The relationship between panicle architecture and genes involved in BRs signaling and biosynthesis

It is well known that there is an interplay between the signaling and biosynthesis of BRs in rice [49]. However, some of these tested BR-related genes, including *RAVL1*, *OsBRI1*, *OsLIC* and *SG1*, did not show conspicuous alterations in *pmm1–1* except for *MDP1* and *DWARF4* (Fig. 8a). A previous study suggested that *OsMDP1* function in BR signal transduction and act as a negative regulator of floral organ development and floral identity in rice [65]. To investigate whether panicle architecture in rice requires *OsMDP1*, a closer examination of panicle morphology with *osmdp1* need to be carried out.

As previously reported, *D11* and *D4* may function redundantly in C-22 hydroxylation for BR biosynthesis [54]. We have verified that overexpression of *D4* under the background of *pmm1–1* mutant could completely rescue the abnormal inflorescence in *pmm1–1* (Fig. 7c). These results indicate that enhancing the expression level of *D4* could complement the BR deficit in *pmm1–1*. However, in plants with gain or loss function of *D4*, no significant variations could be observed in branch and spikelet morphology (Fig. 7c), indicating that *D4* is not crucial to the establishment of panicle morphology in rice. These results suggest that *PMM1* acts on the establishment of rice panicle architecture by regulating BR accumulation, while *D4* might contribute to vegetative development preferentially.

## Conclusion

We have identified a new allele of *D11, PMM1,* encoding a cytochrome P450 protein involved in BR biosynthesis pathway. A large fragment deletion in *pmm1–1* caused an obvious change in rice panicle morphology. Our research revealed that BR biosynthesis is required for the primordial initiation of branches and spikelets during the young panicle development. Thus, *PMM1* determines the inflorescence architecture of rice by controlling brassinosteroid biosynthesis.

## Methods

### Plant materials and growth conditions

All rice plants used in this study were *japonica* (*O. sativa ssp. geng*) variety Zhonghua 11 (ZH11). Three *pmm1* mutants were obtained by screening the enhancer trap mutant library [60]. Rice plants were cultivated in the experimental field of Huazhong Agriculture University in the normal growing season in Wuhan, China (latitude 30.5°N, 15 m above sea level; average daily temperature approximately 28 °C).

### Plasmid construction and rice transformation

To prepare the complementation vector, we digested ZH11 BAC clone OSJNBa0020J04 (kindly provided by Luo Meizhong) with *EcoR*I and *Xba*I, and a 6.2-kb genomic DNA fragment containing the entire *PMM1* coding region and the 1600-bp upstream and 800-bp downstream sequences was inserted into pCAMBIA2301. The empty vector pCAMBIA2301 (http://www.cambia.org/daisy/cambia/585) was used as a negative control. For overexpression of *PMM1/D11* or *OsDWARF4*, full-length cDNA of *PMM1/D11* or *DWARF4* was cloned into the pU1301 vector, which was then electroporated into the Agrobacterium tumefaciens strain EHA105, and finally transformed into rice callus to generate transgenic plants.

### Scanning electron microscopy

For scanning electron microscopy, young panicles at different developmental stages from WT and *pmm1–1* mutants were dissected, subsequently fixed with a blade, and immediately placed in 70% ethanol, 5% acetic acid, and 3.7% formaldehyde for 24 h at 4 °C overnight. Tissues were dehydrated through an ethanol series of 25 to 100% and dried. Following ethanol dehydration, the samples were critical point dried, sputter coated with gold in an E-100 ion sputter, and then observed under a scanning electron microscope (S570, Hitachi, Tokyo, Japan).

### RNA extraction and gene expression analysis

Total RNA was extracted from frozen samples using TRIZOL reagent (Invitrogen). After RNase-free DNase I treatment, the first-strand cDNA was synthesized from 4 μg of total RNA with oligo (dT)_15_ as the primer, using a reverse transcription kit (Invitrogen). qRT-PCR was carried out using ABI7500 real-time PCR system with the SYBR Premix Ex Taq (TaKaRa) following the manufacturer’s instructions. The rice UBIQUITIN5 gene was used as an internal control. Gene expression level was determined from three independent replicates, each consisting of three plants, and three technical replicates per tissue sample were analyzed. The primers for real-time PCR are listed in Additional file 5: Table S3.

### BR test

The WT and *pmm1–1* mutant were used for BR induction experiments. Rice seeds were sterilized with 0.1% HgCl_2_ and then grown on 1% agar medium containing half-strength MS medium in complete darkness at 28 °C for 10 days. For BL (Wuhan DingGuo Biotech. Co. Ltd) induction of shoot elongation, rice seeds were grown on 1% agar medium containing half-strength MS medium and various concentrations (10^− 7^ M and 10^− 6^ M) of BL and incubated at 28 °C under continuous light. After 2 weeks, the length of coleoptile and the appearance of hypocotyl were measured. A total of 45 plants were used for each treatment.

### In situ hybridization

RNA in situ hybridization was performed as described previously [66]. The probe was PCR-amplified from ZH11 using primers in situ-PMM1 F/R (Additional file 5: Table S3). The sense and antisense probes were then transcribed in vitro from the T7 or SP6 promoter with polymerase using a digoxigenin RNA labeling kit (Roche).

## Additional files

Additional file 1: **Table S1.** Primers of SSR markers for mapping *PMM1*. (DOCX 16 kb)

Additional file 2: **Table S2.** Predicted genes in the region containing *PMM1*. (DOCX 17 kb)

Additional file 3: **Figure S1.** Sequence alignment of amino acids of OsDWARF4 and PMM1/OsDWARF11. (**a**) Sequence alignment of OsDWARF4 (CYP90B2) and OsDWARF11 **(**CYP724B1) from rice using the MEGA5.2 analysis tool. Identical and similar amino acid residues are shaded in black and gay, respectively. (**b**) Relative expression of *OsDWARF4* in the root, stem, leaf and sheath and in developing panicles with 1, 2, 3.5, 4.5, 6.5, 8, 10 and 16.5-cm lengths before heading. Rice *UBIQIUTIN5* was used as an internal control. Data are presented as means ± SE (*n* = 3). Significant at **P < 0.01. (DOCX 3903 kb)

Additional file 4: **Figure S2.** Phenotype comparison of lamina joint inclination and plant height between the wild type (WT) and *pmm1–1* plants. **(a)** A close-up view of flag leaf angles of the WT and *pmm1–1* plants. **(b)** A close-up view of the secondary leaf angles the WT and *pmm1–1* plants. **(c)** Measurements of flag leaf angles of the WT and *pmm1–1* plants. **(d)** Measurements of the secondary leaf angles of WT and *pmm1–1* plants. **(e)** The panicle and culm of the WT (left) and *pmm1–1* (right). **(f)** The internodes and panicle of the WT (left) and *pmm1–1* (right). **(g)** Measurements of the length between the WT and *pmm1–1* plants. **(h)** The expression levels of genes associated with lamina joint inclination. **(i)** The expression levels of BR-related genes associated with internode elongation. Rice *UBIQIUTIN5* was used as an internal control. Data are presented as means ± SE (*n* = 3). Significant at **P < 0.01. (DOCX 5072 kb)

Additional file 5: **Table S3.** Primers for qRT-PCR, genotyping, and plasmid construction. (DOCX 18 kb)

## Abbreviations

BM: Branch meristem; SM: Spikelet meristem
BRs: Brassinosteroids
*D11*: *DWARF 11*
*D4*: *DWARF 4*
PMM1: Panicle Morphology Mutant 1
SAM: Shoot apex meristem
