# Supplementary material for: Panicle Morphology Mutant 1 (PMM1) determines the inflorescence architecture of rice by controlling brassinosteroid biosynthesis
Source: BMC Plant Biol. 2018 Dec 12;18:348. doi: 10.1186/s12870-018-1577-x (PMC6291947; doi:10.1186/s12870-018-1577-x)
Supplement: Supplementary file 1 — Table S1. Primers of SSR markers for mapping PMM1. (DOCX 16 kb) [file 12870_2018_1577_MOESM1_ESM.docx]

**Table S1. Primers of SSR markers for mapping *PMM1*.**

| **Marker** | **Forward primer (5'-3')** | **Reverse primer (5'-3')** |
| --- | --- | --- |
| RM17079 | GTGTGAATCTGGACATACCCTAAGC | TTTCTTCCTCCTCATCTGGTTGC |
| RM17108 | CAACTACCGGCCTAAAGCATAGC | GGTGTTTAGGAAGGTGAGGAGAGG |
| RM3866 | TGGTCATCTACCAGAGCAAATTCC | CCGTCATGTCATTGATCTTCTTGC |
| X4 | GCATGCTTTCTTTATATGGAGTC | CTCTTAGTACCCCAGTTTGAATT |
| RM17121 | TGAGATCGAGAGCGTAGGTAGCC | GATATCAGCAGCGACAAGAAAGG |
| RM17131 | CGCGTCGTCAAGAACTCACG | ATCCTCATTGTGCCGTCAAGC |
| RM17140 | AACCGAGCTTCAGAGCTGTAATGG | AAGGTGAGGAGCTGGAGCTTGG |
| RM17143 | CAGCACCCTGACAGTTAAACACTCC | CAATGACCCTGTCATGGATGG |
| RM3839 | ATGCATGTGATGCCAAGAGTGG | GAAAGCACACTGCACACATACCC |
