# Supplementary material for: Panicle Morphology Mutant 1 (PMM1) determines the inflorescence architecture of rice by controlling brassinosteroid biosynthesis
Source: BMC Plant Biol. 2018 Dec 12;18:348. doi: 10.1186/s12870-018-1577-x (PMC6291947; doi:10.1186/s12870-018-1577-x)
Supplement: Supplementary file 2 — Table S2. Predicted genes in the region containing PMM1. (DOCX 17 kb) [file 12870_2018_1577_MOESM2_ESM.docx]

**Table S2. Predicted genes in the region containing *PMM1*.**

| **Number** | **Gene Locus** | **predicted function** |
| --- | --- | --- |
| 1 | LOC_Os04g39260 | CAF1 family ribonuclease containing protein |
| 2 | LOC_Os04g39270 | putative indole-3-glycerol phosphate synthase |
| 3 | LOC_Os04g39280 | ATPOT1 |
| 4 | LOC_Os04g39290 | heavy metal transport/detoxification protein |
| 5 | LOC_Os04g39300 | heavy metal transport/detoxification protein |
| 6 | LOC_Os04g39310 | retrotransposon protein |
| 7 | LOC_Os04g39320 | expressed protein |
| 8 | LOC_Os04g39330 | hypothetical protein |
| 9 | LOC_Os04g39340 | hypothetical protein |
| 10 | LOC_Os04g39350 | heavy metal associated domain containing protein |
| 11 | LOC_Os04g39360 | heavy metal transport/detoxification protein |
| 12 | LOC_Os04g39370 | heavy metal associated domain containing protein |
| 13 | LOC_Os04g39380 | heavy metal transport/detoxification protein |
| 14 | LOC_Os04g39390 | retrotransposon protein |
| 15 | LOC_Os04g39400 | retrotransposon protein |
| 16 | LOC_Os04g39410 | pentatricopeptide |
| 17 | LOC_Os04g39420 | 6-phosphofructokinase 2 |
| 18 | LOC_Os04g39430 | cytochrome P450 |
| 19 | LOC_Os04g39440 | ras-related protein |
| 20 | LOC_Os04g39444 | LSM domain containing protein |
| 21 | LOC_Os04g39450 | expressed protein |
