# Supplementary material for: Panicle Morphology Mutant 1 (PMM1) determines the inflorescence architecture of rice by controlling brassinosteroid biosynthesis
Source: BMC Plant Biol. 2018 Dec 12;18:348. doi: 10.1186/s12870-018-1577-x (PMC6291947; doi:10.1186/s12870-018-1577-x)
Supplement: Supplementary file 3 — Figure S1. Sequence alignment of amino acids of OsDWARF4 and PMM1/OsDWARF11. (a) Sequence alignment of OsDWARF4 (CYP90B2) and OsDWARF11 (CYP724B1) from rice using the MEGA5.2 analysis tool. Identical and similar amino acid residues are shaded in black and gay, respectively. (b) Relative expression of OsDWARF4 in the root, stem, leaf and sheath and in developing panicles with 1, 2, 3.5, 4.5, 6.5, 8, 10 and 16.5-cm lengths before heading. Rice UBIQIUTIN5 was used as an internal control. Data are presented as means ± SE (n = 3). Significant at **P < 0.01. (DOCX 3903 kb) [file 12870_2018_1577_MOESM3_ESM.docx]

**
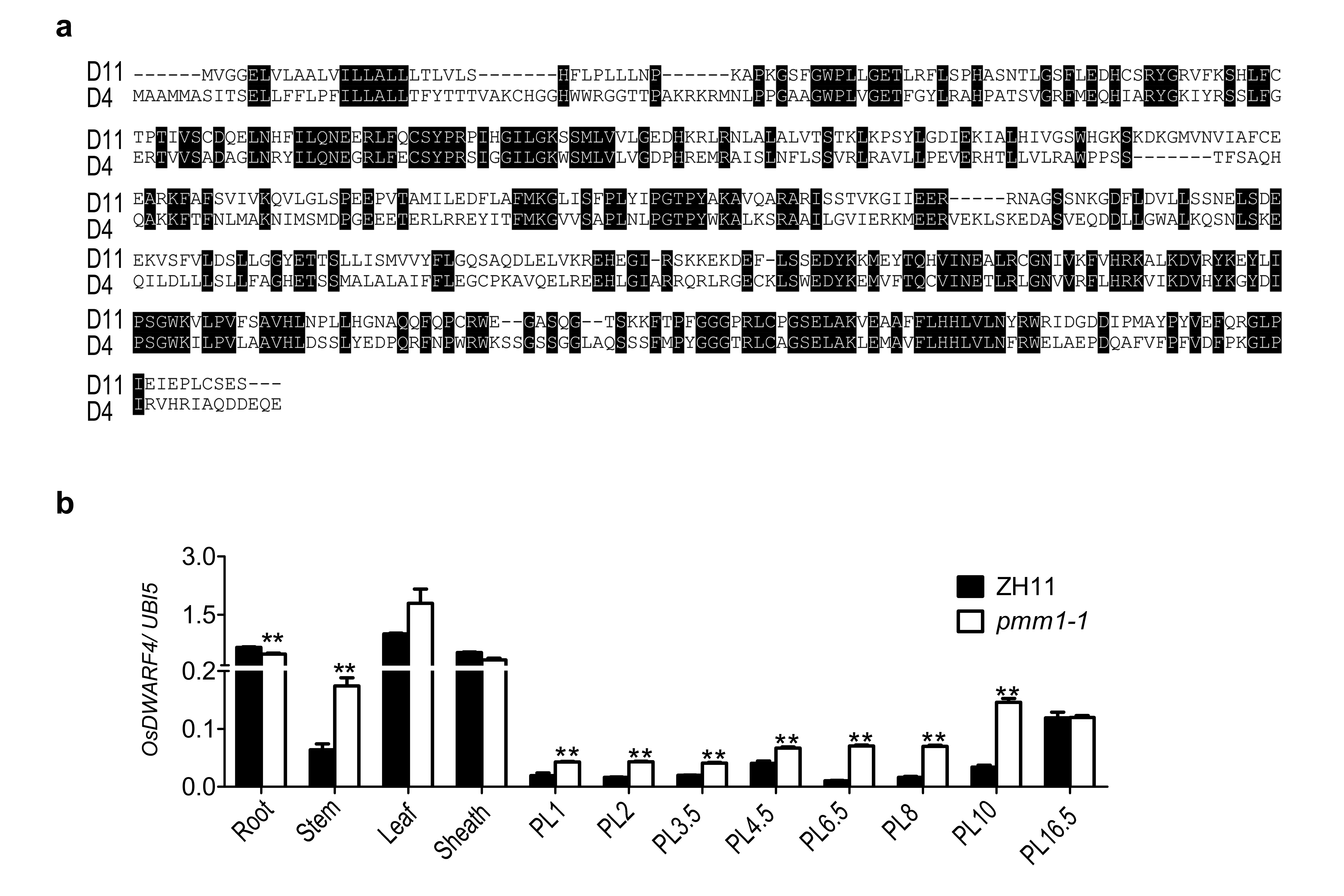
**

**Figure S1. Sequence alignment of amino acids of OsDWARF4 and PMM1/OsDWARF11.**

(a) Sequence alignment of OsDWARF4 (CYP90B2) and OsDWARF11 **(**CYP724B1) from rice using the MEGA5.2 analysis tool. Identical and similar amino acid residues are shaded in black and gay, respectively. (b) Relative expression of *OsDWARF4* in the root, stem, leaf and sheath and in developing panicles with 1, 2, 3.5, 4.5, 6.5, 8, 10 and 16.5-cm lengths before heading. Rice *UBIQIUTIN5* was used as an internal control. Data are presented as means ± SE (n=3). Significant at **P< 0.01.
