# Supplementary material for: Panicle Morphology Mutant 1 (PMM1) determines the inflorescence architecture of rice by controlling brassinosteroid biosynthesis
Source: BMC Plant Biol. 2018 Dec 12;18:348. doi: 10.1186/s12870-018-1577-x (PMC6291947; doi:10.1186/s12870-018-1577-x)
Supplement: Supplementary file 4 — Figure S2. Phenotype comparison of lamina joint inclination and plant height between the wild type (WT) and pmm1–1 plants. (a) A close-up view of flag leaf angles of the WT and pmm1–1 plants. (b) A close-up view of the secondary leaf angles the WT and pmm1–1 plants. (c) Measurements of flag leaf angles of the WT and pmm1–1 plants. (d) Measurements of the secondary leaf angles of WT and pmm1–1 plants. (e) The panicle and culm of the WT (left) and pmm1–1 (right). (f) The internodes and panicle of the WT (left) and pmm1–1 (right). (g) Measurements of the length between the WT and pmm1–1 plants. (h) The expression levels of genes associated with lamina joint inclination. (i) The expression levels of BR-related genes associated with internode elongation. Rice UBIQIUTIN5 was used as an internal control. Data are presented as means ± SE (n = 3). Significant at **P < 0.01. (DOCX 5072 kb) [file 12870_2018_1577_MOESM4_ESM.docx]

**
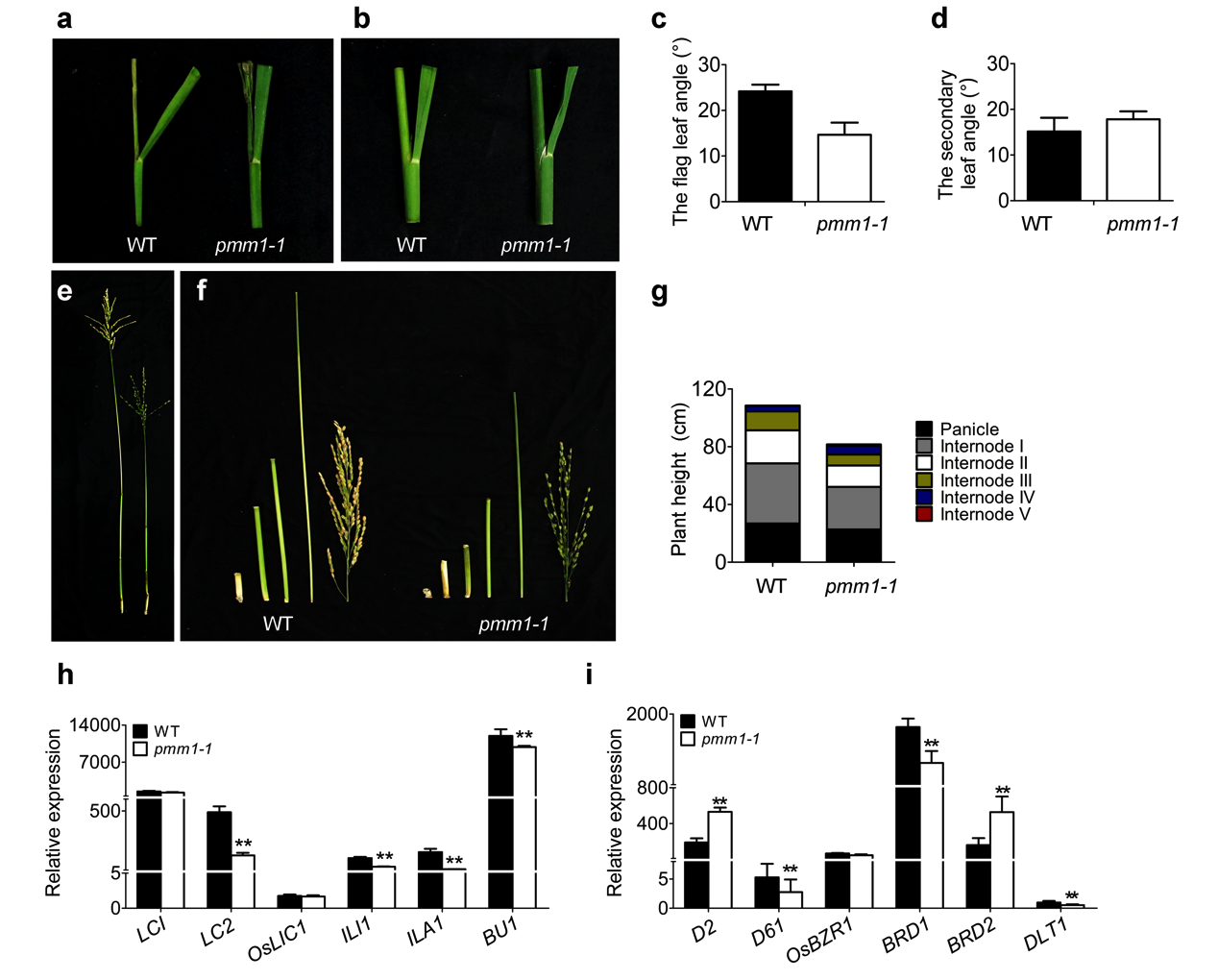
**

**Figure S2. Phenotype comparison of lamina joint inclination and plant height between the wild type (WT) and *pmm1-1* plants.**

**(a)** A close-up view of flag leaf angles of the WT and *pmm1-1* plants. **(b)** A close-up view of the secondary leaf angles the WT and *pmm1-1* plants. **(c)** Measurements of flag leaf angles of the WT and *pmm1-1* plants. **(d)** Measurements of the secondary leaf angles of WT and *pmm1-1* plants. **(e)** The panicle and culm of the WT (left) and *pmm1-1* (right). **(f)** The internodes and panicle of the WT (left) and *pmm1-1* (right). **(g)** Measurements of the length between the WT and *pmm1-1* plants. **(h)** The expression levels of genes associated with lamina joint inclination. **(i)** The expression levels of BR-related genes associated with internode elongation. Rice *UBIQIUTIN5* was used as an internal control. Data are presented as means ± SE (n=3). Significant at **P< 0.01
