# Supplementary material for: Panicle Morphology Mutant 1 (PMM1) determines the inflorescence architecture of rice by controlling brassinosteroid biosynthesis
Source: BMC Plant Biol. 2018 Dec 12;18:348. doi: 10.1186/s12870-018-1577-x (PMC6291947; doi:10.1186/s12870-018-1577-x)
Supplement: Supplementary file 5 — Table S3. Primers for qRT-PCR, genotyping, and plasmid construction. (DOCX 18 kb) [file 12870_2018_1577_MOESM5_ESM.docx]

**Table S3. Primers for qRT-PCR, genotyping, and plasmid construction.**

| **Primer** | **Forward primer (5'-3')** | **Reverse primer (5'-3')** |
| --- | --- | --- |
| qRT-PMM1 | GTGCTCAATTATAGATGGAGAATCG | TCGATTTCTATGGGCAGACCTCT |
| qRT-MDP1 | TTATTGACCGGTACAACTCGCA | TCCAGTCCATCGATCTCATCC |
| qRT-BU1 | GTAGCCAGCTTGATCTCATCTC | GGGACGACTCTACTGCATCA |
| qRT-ILI1 | TTCTCTCCAAGCTTCAGGCCCT | TGCACGTCTCCTGCAAAACCCT |
| qRT-RAVL1 | GCACTCCTCGTTGGATCTCGAT | GCAGCAGACGGAAGATGGATT |
| qRT-OsBRI1 | CTTGGTTCCCTTGCCACATTT | CCTCATTCTTCAGCCTCCGC |
| qRT-LIC | GGAGTTTCGAGCGTATCTGGAA | TGGACAGAGGAAGCAGGAGACT |
| qRT-SG1 | CCCAGATGGATCTTGGAGGT | GGCAGACAGCAAGCTGAAAG |
| qRT-DWARF4 | GAGATGGTTTTCACGCAATGTG | ACCCTTGTAGTGCACGTCCTTG |
| qRT-LAX1 | TGCCTCCTTTGCCTTGCT | TGGACATTGCACACCGAGTAG |
| qRT-FZP | CTCCGACTCCTACTCTCCATTCA | CAATGCCCGGTGCAACTC |
| qRT-LAX2 | GGAGGGTGACAGTAAGGTTGCT | CCTCTGATTCATCTTCCAATCCA |
| qRT-MCM2 | AAGTTGGCAAAAGATCCACGG | CCCCCAAACATAGCTAGTGCAA |
| qRT-MCM3 | TTCATGCGTCACTAAATGCGAG | TGAATCTGGAAGCCCAATGTTC |
| qRT-CYCB2 | CTCAAGGCTGCACAATCTGACA | GCATTGACGGCTGGAATTTG |
| qRT-CYCB2.1 | TTGCCAGCAGTGGACAAAGAC | CCGCCTTCTGGTGGAAATCTA |
| qRT-CYCIaZm | CACTCTCAAGCACCACACTGGA | ACAACCCTCAGCTTGCTCTCAG |
| qRT-CYCA2.3 | GTTTCGGTTGACGAGACGATGT | CGCTGCAAGGAACCTAGAACTG |
| qRT-GAPDH | CGACCCGTTCATCACCACCGAC | AGCTAGCAGCCCTTCCACCTCTCCA |
| In situ-PMM1 | CCGGTCACTGCCATGATACTT | GAGCCTTCCTGTGGACAA ACT |
| PMM1 | ACAATGCAAGAAAGAAATACCCCTA | GCAAGTAACAACATGATGATGATGG |
| Gal4 | GCCTCAAGAAGCTCAAGTGC | GGCATCGGTAAACATCTGCT |
| PMM1-OE | AGTGGTACCGAGATAAGATGAGCAG | AGTGGATCCTCACTGACTAATCCTCT |
| DWARF4-OE | AGTGGTACCATACTCTTGTAATTTGGCGAGCA | AGTGGATCCATCCTTTCCTGATCACATCTACACA |
